# Cas9 interaction with the tracrRNA nexus modulates the repression of type II-A CRISPR-cas genes

**DOI:** 10.1093/nar/gkae597

**Published:** 2024-07-12

**Authors:** Hyejin Kim, Luciano A Marraffini

**Affiliations:** Laboratory of Bacteriology, The Rockefeller University, 1230 York Ave, New York, NY 10065, USA; Weill Cornell/Rockefeller/Sloan Kettering Tri-Institutional MD-PhD Program, New York, NY, USA; Laboratory of Bacteriology, The Rockefeller University, 1230 York Ave, New York, NY 10065, USA; Howard Hughes Medical Institute, The Rockefeller University, 1230 York Ave, New York, NY 10065, USA

## Abstract

Immune responses need to be regulated to prevent autoimmunity. CRISPR-Cas systems provide adaptive immunity in prokaryotes through the acquisition of short DNA sequences from invading viruses (bacteriophages), known as spacers. Spacers are inserted into the CRISPR locus and serve as templates for the transcription of guides used by RNA-guided nucleases to recognize complementary nucleic acids of the invaders and start the CRISPR immune response. In type II-A CRISPR systems, Cas9 uses the guide RNA to cleave target DNA sequences in the genome of infecting phages, and the tracrRNA to bind the promoter of *cas* genes and repress their transcription. We previously isolated a Cas9 mutant carrying the I473F substitution that increased the frequency of spacer acquisition by 2–3 orders of magnitude, leading to a fitness cost due to higher levels of autoimmunity. Here, we investigated the molecular basis underlying these findings. We found that the I473F mutation decreases the association of Cas9 to tracrRNA, limiting its repressor function, leading to high levels of expression of *cas* genes, which in turn increase the strength of the type II-A CRISPR-Cas immune response. We obtained similar results for a related type II-A system, and therefore our findings highlight the importance of the interaction between Cas9 and its tracrRNA cofactor in tuning the immune response to balanced levels that enable phage defense but avoid autoimmunity.

## Graphical Abstract

**Graphical Abstract:**
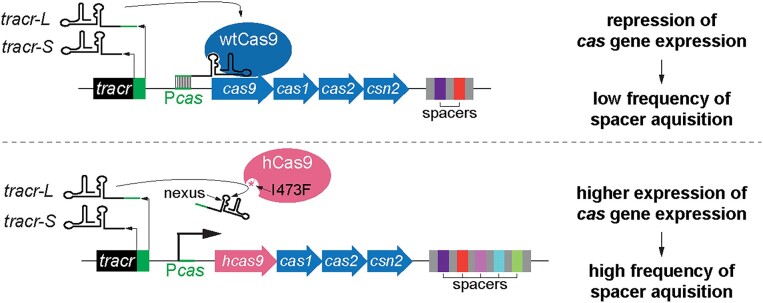

## Introduction

Immune systems must recognize and eliminate rapidly evolving threats to protect the host population. Present in ∼50% of bacterial and ∼90% of archaeal genomes (1,2), clustered, regularly interspaced, short, palindromic repeats (CRISPR) loci and their associated genes (*cas*) defend bacteria from invading phages (3) and plasmids (4). The hallmark of CRISPR immunity is the acquisition of immunological memories in a process called adaptation. During CRISPR adaptation, short DNA sequences from foreign invaders known as spacers (5–7) are acquired and integrated into the CRISPR locus in between the repeats (3). The spacers serve as templates for the generation of short RNA guides known as CRISPR RNAs (crRNAs) that associate with Cas nucleases to recognize and cleave complementary sequences present in invader nucleic acids to provide immunity (8–13).

CRISPR-Cas systems can be classified into six types based on their *cas* gene content (2,14). In type II-A CRISPR-Cas systems, the crRNA-guided nuclease Cas9 generates dsDNA breaks on the invader's genome to destroy phages and plasmids (13), an activity that requires an RNA cofactor known as the tracrRNA (15). In addition to a sequence with complementarity to the spacer of the crRNA guide, known as the protospacer, Cas9 targets contain a downstream protospacer-adjacent motif (PAM) (16,17). In the case of *Streptococcus pyogenes* SF370 Cas9, the optimal motif is 5′-NGG-3′, with 5′-NAG-3′ supporting lower levels of DNA targeting (18). In addition to DNA cleavage at the target sites complementary to the crRNA, Cas9 can use the tracrRNA as a guide to bind the promoter of the type II-A *cas* operon (P*cas*) and repress its transcription (19). Some bacterial species, including *S. pyogenes*, produce a longer form of the tracrRNA, known as *tracr-L*, with an extended sequence complementary to a region within P*cas* that is followed by a NGG PAM.

A previous study from our lab subjected *S. pyogenes cas9* to mutagenic PCR amplification to screen for variants with increased targeting efficiency against protospacers followed by an NAG PAM (20). This experiment led to the identification of ‘hyper’ Cas9 (hCas9), a mutant harboring a I473F substitution that not only enhances NAG targeting but also spacer acquisition by 2–3 orders of magnitude. Residue I473 is part of a pocket that accommodates a hairpin within the tracrRNA known as the nexus (15,21,22) and therefore it is not immediately clear how the mutation of this residue to phenylalanine can cause these phenotypes. Here we investigated the molecular mechanism underlying the enhanced type II-A CRISPR-Cas response mediated by hCas9. We found that the I473F mutation decreases Cas9 binding to *tracr-L* to relieve the repression of the *cas* operon and increase transcription of the type II-A *cas* genes. This result is in line with previous reports describing experiments in which enhanced spacer acquisition into the type II-A CRISPR array was achieved through either over-expression of the genes required for spacer integration *cas1*–*cas2*–*csn2* (23,24) or by introducing mutations in the tracrRNA locus that prevent the generation of *tracr-L* (19). We also investigated the nexus-interacting pocket of Cas9 and found that substitutions of other residues within this pocket display the same phenotypes of hCas9, results that highlight a key role of this pocket in the activity of Cas9 as a P*cas* repressor. Finally, we explored the role of I473, which is highly conserved, in Cas9 nucleases from other organisms, demonstrating the importance of this residue in the type II-A CRISPR-Cas response.

## Materials and methods

### Bacterial strains and growth conditions

Growth of *S. aureus* was carried out in brain-heart infusion broth (BHI) at 37°C with shaking at 220 RPM. The media was supplemented as needed with chloramphenicol at 10 μg/ml for maintenance of pC194-derived plasmids (25), erythromycin at 10 μg/ml for maintenance of pE194-derived plasmids (26), and spectinomycin at 250 μg/ml for maintenance of pLZ12-derived plasmids (27). For bacteriophage infection, the media was supplemented with 5 mM CaCl2 to facilitate adsorption. The bacterial strains and phage used in this study can be found in Supplemental Information.

### Plasmid construction

All the plasmids used in this study can be found in Supplementary Table 1, and the DNA oligos used to construct them via Gibson assembly in Supplementary Table 2. The type II-A CRISPR-Cas system of *S. mutans* NN2025 was cloned using Gibson assembly using pLZ-12 and pJM75 with primers HK391, HK392, HK393 and HK394. pJM75 was initially constructed using pC194 and *S. mutans* NN2025 genomic DNA as templates with primers JM386, JM389, JM511 and JM512. 100 ng of the largest dsDNA fragment for assembly was combined with equimolar volumes of the smaller fragment and brought to 10 uL total in dH2O. Samples were added to 10 ul of Gibson Assembly mix (NEB) and incubated at 50°C for 1 h. Samples were drop-dialyzed for 45 min and electroporated into electrocompetent *S. aureus* RN4220 cells (28).

### Total RNA extraction from overnight cultures

Overnight cultures were launched from glycerol stocks, spun down, resuspended in 150uL1XPBS and 100 μg/ml lysostaphin and incubated at 37°C for 20 min. 450 μl Trizol and 600 μl 200 proof ethanol were added to the cell lysate and samples were vortexed. RNA was extracted using the Direct-Zol Miniprep Plus spin column (Zymo) and treated with TURBO DNase (ThermoFisher) according to the manufacturers’ protocols.

### Total RNA extraction from early log phase cultures

Overnight cultures were launched from glycerol stocks. The next day, the cultures were diluted 1:100 in BHI supplemented with antibiotic. The cultures were outgrown for about 1 hour 10 min (OD 0.2–0.4) and then normalized for optical density before being spun down. Pellets were resuspended in 150uL1XPBS and 100 μg/ml lysostaphin and incubated at 37°C for 20 min. 450 μl Trizol and 600 μl 200 proof ethanol were added to the cell lysate and samples were vortexed. RNA was extracted using the Direct-Zol Miniprep Plus spin column (Zymo) and treated with TURBO DNase (ThermoFisher) according to the manufacturers’ protocols.

### RT-qPCR

100 ng of purified total RNA was reverse transcribed with SuperScript IV Reverse Transcriptase (ThermoFisher) according to the manufacturer's protocol. 40 ng of cDNA for each sample was then used for qPCR using PowerUp SYBR Green Master Mix (Life Technologies). The fast cycling mode for standard quantification was selected on the QuantStudio 3 Real-Time PCR System (Applied Biosystems). Cycling conditions were as follows: 50°C for 2 min, 95°C for 2 min, 40 cycles of [60°C 30 s], followed by a melt curve: 60–95°C, incrementing 0.15°C every 1 s. RNA abundance was calculated by the ΔΔCt method. RNA abundance was normalized to Ct values for a host-specific primer set targeting the housekeeping *rho* gene to control for total RNA content. qPCR primer sequences are provided in Supplemental Information.

### Promoter activity

Cells encoding *S. pyogenes* type II-A CRISPR-Cas on pC194 backbones were transformed with a second pE194-derived plasmid (Erm resistance cassette) harboring a transcriptional GFP fusion to the indicated promoter. For *S. mutans*, cells encoding type II-A CRISPR-Cas on pLZ12 backbones were transformed with a second pLZ12-derived plasmid (resistance cassette swapped for Cm) harboring a transcriptional GFP fusion to the indicated promoter. Overnight cultures were launched from these colonies. 200 μl of overnight cultures were spun down in a 1.5 ml Eppendorf tube at 6000 rpm for 1 min. Cell pellets were resuspended in 1 ml of 1× PBS and 150 μl were transferred into a clear, flat-bottomed 96-well plate (Grenier 655 180). Promoter activity was measured as the ratio of fluorescence (excitation wavelength = 485 nm; emission wavelength = 535 nm) to absorbance (at 600 nm) and normalized to an empty vector control.

### Top agar infections and PCR analysis of CRISPR arrays

Overnight cultures were launched from glycerol stocks. The next day, the cultures were diluted 1:100 in BHI supplemented with antibiotic and 5 mM CaCl_2_. The cultures were outgrown for about 1 hour 45 min and then normalized for optical density before being infected with phage of appropriate MOI. Cells were suspended in 50% heart infusion agar (HIA) supplemented with 5 mM CaCl_2_ and antibiotic. Plates were incubated overnight at 37^o^C and surviving colonies were enumerated the next day. A subset of surviving colonies was resuspended in 30 μl of colony lysis buffer (250 mM KCl, 5 mM MgCl_2_, 50 mM Tris–HCl at pH 9.0 and 0.5% Triton X-100) supplemented with 200 ng/μl of lysostaphin. The reactions were incubated for 20 min at 37°C and then for 10 min at 98°C in a thermocycler. 2 μl of each reaction was used to PCR amplify the CRISPR array with Phusion master mix (Thermofisher). Cycling was performed under the following conditions: 98°C for 30 s, 34 cycles of [98°C 10 s, 58°C 20 s, 72°C for 30s/kb], 72°C 5 min, hold at 12°C. Primer sequences are provided in Supplementary Table 2. The PCR products were analyzed on a 2% agarose gel stained with ethidium bromide and imaged with FluorChem HD2 (Protein simple).

### Plaque formation assay

Ten-fold serial dilutions of bacteriophage (3 μl each) were spotted on a layer of *S. aureus* cells suspended in 50% brain-heart infusion agar (BHI) supplemented with 5 mM CaCl_2_ and the appropriate antibiotic as needed. The plates were incubated overnight at 37°C after drying at room temperature for 20 min and plaque-forming units (PFU) were enumerated the next day. Efficiency of plaquing was calculated by normalizing plaque forming units/μl to an empty vector control.

### Growth curves and PCR analysis of CRISPR arrays

Overnight cultures were launched from glycerol stocks. The next day, the cultures were diluted 1:100 in BHI and 5 mM CaCl_2_. The cultures were outgrown for about 1 h 10 min and then normalized for optical density before being infected with phage of appropriate MOI. 140 μl of cultures were seeded in a flat-bottom 96-well plate (Cellstar). Absorbance at 600 nm was recorded every 10 min for 24 h in a microplate reader (TECAN Infinite 200 PRO). After 24 h, 2 μl from the pCRISPR cultures were resuspended in 30 μl of colony lysis buffer (250 mM KCL, 5 mM MGCl2, 50 mM Tris–HCl at pH 9.0 and 0.5% Triton X-100) supplemented with 200 ng/μl of lysostaphin. The reactions were incubated for 20 min at 37 °C and then for 10 min at 98 °C in a thermocycler. 1 μl of each reaction was used to amplify the CRISPR array with Phusion master mix (Thermofisher). Cycling was performed under the following conditions: 98°C for 30 s, 34 cycles of [98°C 10 s, 58°C 20 s, 72°C for 30 s/kb], 72°C 5 min, hold at 12°C. Primer sequences are provided in Supplemental Information. The PCR products were analyzed on a 2% agarose gel stained with ethidium bromide and imaged with FluorChem HD2 (Protein simple).

### Western blot

Overnight cultures of 3xFLAG-tagged Cas9 (N-terminal tags) natively expressed in pC194 backbones were launched from glycerol stocks. The next day, the cultures were spun down, resuspended in 150 ul 1× PBS and 100 μg/ml lysostaphin and incubated at 37°C for 20 min. Lysates were mixed 1:1 with 2× Laemmli (Bio Rad) supplemented with BME, boiled at 98°C for 5 min, and run on 4–20% Mini-PROTEAN TGX Precast Protein Gels (Bio Rad). Transferred proteins were probed for 2 h at room temperature with anti-FLAG M2 antibody in PBST (1:4000 dilution) (Sigma) for Cas9 (∼160 kDa) and with anti- RNA polymerase β antibody in PBST (1:4000) (BioLegend) for blotting control RNA polymerase β (∼150 kDa). Anti-mouse IgG secondary antibody, HRP in PBST (Invitrogen) was used to prepare the blots for visualization with Clarity Western ECL Substrate (Bio Rad). Blots were imaged with ImageQuant800 (Amersham).

### Quantification of Cas9-bound RNA

Overnight cultures of 3xFLAG-tagged Cas9 natively expressed in pC194 backbones and containing the first natural spacer present in *S. pyogenes* were launched from glycerol stocks. The next day, the cultures were diluted 1:100 in 100 ml BHI supplemented with appropriate antibiotic and outgrown for about 2 h to OD ∼1. After cultures were normalized, cells were pelleted and resuspended in 20 ml TBS (50 mM Tris–HCl 150 mM NaCl, pH 7.4) supplemented with 10 mM MgCl_2_, 100 μg/ml lysostaphin, and cOmplete protease inhibitor (Sigma-Aldrich). Cells were lysed with needle sonication. 500 μl of FLAG resin was prepared per sample and washed with TBS (5 CVs). Insoluble fraction was pelleted and the remaining soluble lysate was applied to the resin under gravity flow with multiple passes. The column was washed with TBS (10–20 CVs) to remove unbound proteins and 3xFLAG-tagged Cas9 was eluted by competition with 100 μg/ml 3xFLAG peptide. Samples were normalized to total protein and RNA was extracted using the Direct-Zol Miniprep Plus spin column according to the manufacturers’ protocols and prepared for qPCR as described previously. Primers targeting tracr-L target the 5′ region unique to tracr-L that is not present in the other tracrRNA isoforms. RNA abundance was normalized to Ct values for a primer set targeting the specific crRNA generated. qPCR primer sequences are provided in Supplemental Information.

### Structural analysis of Cas9

Molecular graphics image was produced using the UCSF Chimera package from the Resource for Biocomputing, Visualization, and Informatics at the University of California, San Francisco (29).

### RNA structure prediction

tracrRNA secondary structures were predicted using the ViennaRNA web server (30).

### Cas9 multiple sequence alignment

1000 Cas9 sequences were obtained using SpyCas9 as the input for a PSI-BLAST protein search of the RefSeq Select database on NCBI. These sequences were then subjected to a CLUSTAL Omega alignment via the EMBL-EBI web tool (31). Residue frequencies ignoring gaps within the alignment were analyzed using the Geneious Prime 2022.2.1 software.

### Cas promoter sequence alignment

*S. pyogenes* and *S. mutans* cas promoter sequences were subjected to a local Smith-Waterman alignment with SnapGene 7.2.1 software.

### Statistical analyses

Statistical analyses were performed using GraphPad Prism 10. Error bars for all bar graphs represent the standard deviation of replicates. As necessary, a *t*-test was used to compare means. In each case, an unpaired parametric *t*-test was performed with the assumption that samples come from populations with a Gaussian distribution and the same standard deviation. All presented *P*-values are two-sided.

## Results

### The I473F mutation in Cas9 increases cas gene expression

**Figure 1.**
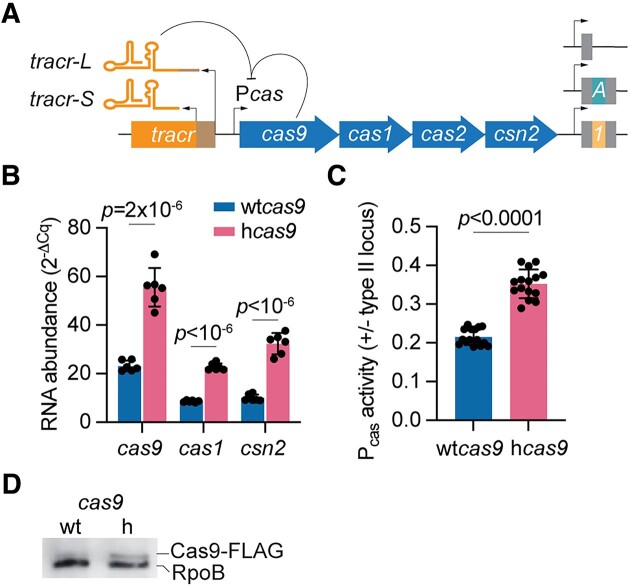
The I473F mutation in Cas9 increases *cas* gene expression. (**A**) Schematic of the type II-A CRISPR-Cas loci of *S. pyogenes*. The tracrRNA locus generates two forms of tracrRNA: *tracr-L* and *tracr-S*. *tracr-L* functions as a single guide RNA that directs Cas9 to bind the *cas* promoter and repress *cas* gene expression. The CRISPR arrays utilized throughout our studies contained either no spacer (single repeat) or a single spacer: ‘1’ is spc1 of the native CRISPR array; ‘A’ is the spacer that matches a target containing an NAG PAM in ϕNM4γ4. (**B**) RNA quantification of *cas* gene transcripts via qRT-PCR normalized to housekeeping gene *rho*. Total RNA was isolated from overnight *S. aureus* cultures harboring pCRISPR containing wtCas9 or hCas9 (I473F). Means of six technical replicates ± SD are reported. (**C**) P*cas* promoter activity measured as the green fluorescence/OD_600_ ratio in cells harboring a plasmid harboring a P*cas*-*gfp* reporter and a second plasmid encoding pCRISPR with wtCas9 or hCas9. Promoter activity was normalized to an empty vector control. Means of five biological replicates (three technical replicates each) ± SD are reported. (**D**) Western blot of protein extracts of staphylococci harboring pCRISPR plasmids expressing FLAG-tagged versions of wtCas9 and hCas9. Detection of RpoB was used as a loading control.

Our lab studies type II-A CRISPR-Cas immunity using a heterologous experimental system where the CRISPR-*cas* locus of *Streptococcus pyogenes* SF370 is cloned into the staphylococcal vector pC194 to generate the pCRISPR plasmid, which was introduced into *Staphylococcus aureus* RN4220 (24) (Fig. 1A). Given that one of the phenotypes of the I473F mutation in Cas9 (hyper-Cas9 or hCas9) is an increase in the frequency of spacer acquisition (20) and that this can also be achieved through over-expression of the type II-A CRISPR-*cas* locus (19,24), we measured the effect of this mutation on *cas* gene expression via RT-qPCR, after normalization to the housekeeping gene *rho*. We found that compared to staphylococci expressing wild-type Cas9 (wtCas9), transcription of the *cas* operon was increased in cells harboring hCas9 (Fig. 1B). To confirm this result, we used a reporter plasmid harboring the *gfp* gene under the control of the type II-A *cas* promoter P*cas* (19). We normalized the number of cells tested using cultures with equivalent optical density at 600 nm, OD_600_, and calculated the ratio of green fluorescence in the presence or absence of pCRISPR plasmids expressing either wtCas9 or hCas9 to repress the P*cas* promoter. We found higher levels of green fluorescence in cultures expressing hCas9 (Fig. 1C), a result that, in addition to the RT-qPCR data, demonstrates that the I473F substitution in Cas9 increases *cas* gene expression in the type II-A CRISPR-*cas* locus, most likely directly affecting the activity of its promoter. Finally, we performed a western blot using protein extracts of staphylococci harboring pCRISPR plasmids expressing FLAG-tagged versions of wtCas9 and hCas9. Consistent with the reporter assays, we found higher levels of hCas9 (Fig. 1D).

### Increased *cas* gene expression enhances the type II-A CRISPR response

**Figure 2.**
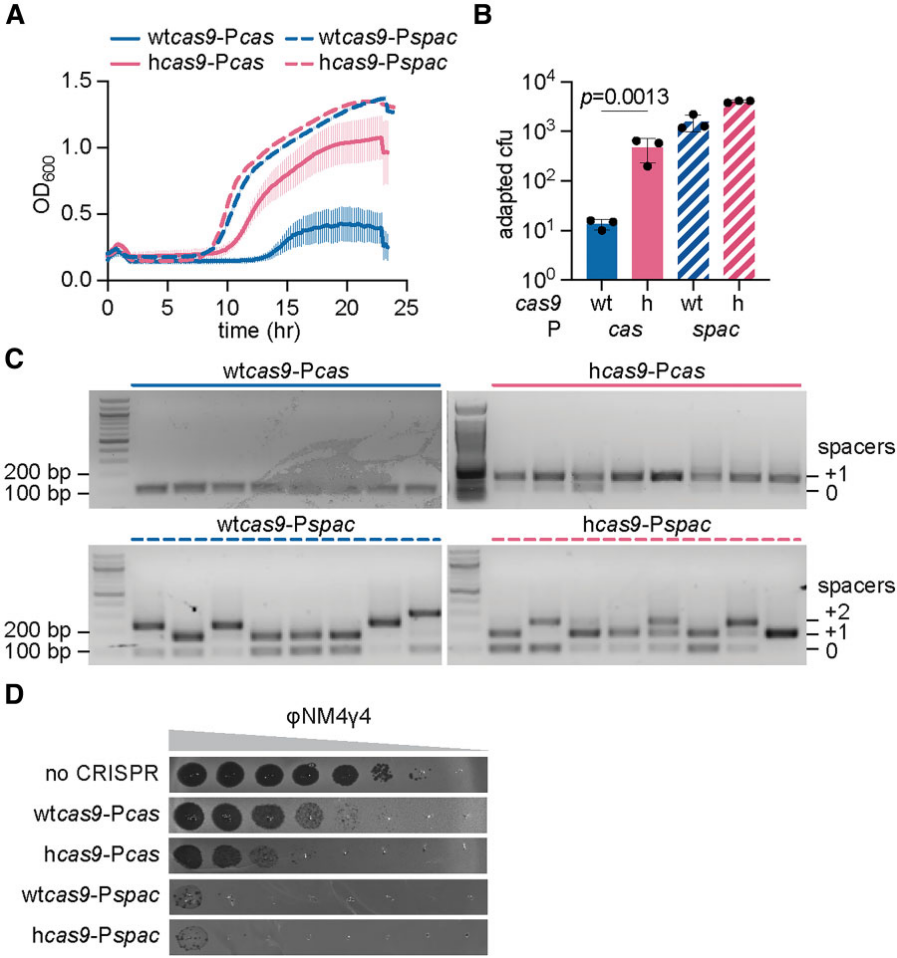
Overexpression of wtCas9 mimics the effects of hCas9 expression. (**A**) Growth of *S. aureus* expressing pCRISPR after infection with ϕNM4γ4 (MOI ∼10), measured as the OD_600_ of the cultures over time. The *cas* genes of the pCRISPR plasmids are under the control of the native P*cas* promoter or the constitutive P*spac* promoter. Means of three biological replicates ± SEM are reported. (**B**) Plasmids harboring naive CRISPR-Cas systems with wtCas9 or hCas9 were infected with ϕNM4γ4 (MOI∼2) on top agar. Surviving colonies with expanded CRISPR arrays were quantified by PCR. Means of three biological replicates ± SD are reported. (**C**) Agarose gel electrophoresis of PCR products obtained after amplification of the CRISPR array using DNA obtained from colonies that formed on top agar after infection with ϕNM4γ4 (MOI∼2) of cells containing pCRISPR whose *cas* genes are under the control of the native P*cas* promoter or the constitutive P*spac* promoter. (**D**) Detection of plaque formation after spotting 10-fold serial dilutions of ϕNM4γ4 on top agar seeded with *S. aureus* carrying an empty vector control or expressing pCRISPR whose *cas* genes are under the control of native P*cas* promoter or the constitutive P*spac* promoter. Plasmids were programmed with spacer ‘A’ to target a region of the viral genome followed by a NAG PAM. Representatives of three biological replicates shown.

Given the above results, we reasoned that the replacement of P*cas* with a strong constitutive promoter should enable staphylococci harboring wt*cas9* to display the phenotypes associated with hCas9 expression. To test this, we exchanged P*cas* in pCRISPR by P*spac*, a strong, constitutive promoter used for gene over-expression in gram-positive bacteria (32). We also introduced this promoter into pCRISPR plasmids carrying h*cas9* as a control to investigate whether the I473F mutation enhances the type II-A CRISPR response independently of the transcription levels of the *cas* genes. First, we used RT-qPCR to determine the effect of the P*spac* promoter on transcription and found that it increased RNA production to even higher degrees than those observed in the presence of hCas9 (Supplementary Figure S1A). Using this plasmid, we then measured spacer acquisition and immunity against NAG targets, the two processes that were shown to be enhanced by the I473F mutation (20). To evaluate spacer acquisition, we used a minimal CRISPR array with no spacer (Fig. 1A) to prevent a phenomenon known as ‘priming’, where existing spacers enhance the acquisition of new ones (33–35). We then infected the different cultures with ϕNM4γ4 phage (24) and followed their growth by measuring OD_600_. As reported before (20), the OD_600_ values drop immediately after infection due to the lysis of the cultures since most staphylococci do not incorporate new spacers that will protect them from the phage. However, the small fraction of cells that do integrate phage sequences into the CRISPR array eventually grow back and mediate an increase in OD_600_ that correlates with the frequency of spacer acquisition in the culture. Therefore, while all cultures are able to acquire new spacers at the end of the experiment (determined by PCR amplification of the CRISPR array, Supplementary Figure S1A), similarly to previous results (20), those expressing hCas9 recover faster (∼9 h after infection) and more strongly (final OD_600_ ∼ 1.0) than bacteria expressing wtCas9 (recovery starts at ∼ 14 h after infection and the culture reaches a final OD_600_ ∼ 0.4), when the *cas* operon is controlled by the native promoter (Figure 2A). In contrast, when the type II-A *cas* operon is under the control of the P*spac* promoter, cells expressing wtCas9 followed a growth pattern more similar to that of cultures harboring hcas9 transcribed from P*cas* (recovery starts at ∼8 h after infection and the culture reaches a final OD_600_ ∼1.2, Figure 2A). We also performed infection in solid media (top agar), where cells that survive form a distinct colony. While most colonies derived from cells harboring the wild-type type II-A CRISPR-*cas* locus developed non-CRISPR phage resistance (most likely contain receptor mutations) (20) and therefore did not show expansion of the CRISPR array, colonies in which transcription of this locus is controlled by the P*spac* promoter acquired one or two new spacers, similarly to the case of cells that expressed hCas9 from the native promoter (Figure 2B, C).

Next, we tested the ability of Cas9 to target a viral protospacer sequence followed by NAG PAM, replacing the natural spacers present in *S. pyogenes* by one designed for this assay (Figs. 1A and Supplementary Figure S1C). We measured phage propagation by quantifying plaque forming units in lawns of staphylococci harboring type II-A CRISPR loci carrying different mutations (Figure 2D). We found that, as reported before (20), in the presence of the native promoter, hCas9 provides 100-fold increase in defense against ϕNM4γ4. In the presence of the P*spac* promoter, wtCas9 targeting increased by four orders of magnitude. Altogether these results are consistent with a model in which increased activity from the native P*cas* promoter is responsible for the enhanced spacer acquisition and NAG targeting phenotypes of hCas9. Importantly, when expressed from the P*spac* promoter, wtCas9 and hCas9 displayed almost identical phenotypes in the three assays described above, a result that demonstrates that the I473F mutation primarily enhances the type II-A CRISPR-Cas response through *cas* over-expression.

### The I473F mutation decreases binding of Cas9 to *tracr-L* to relieve repression of *cas* expression and enhance the type II-A response

**Figure 3.**
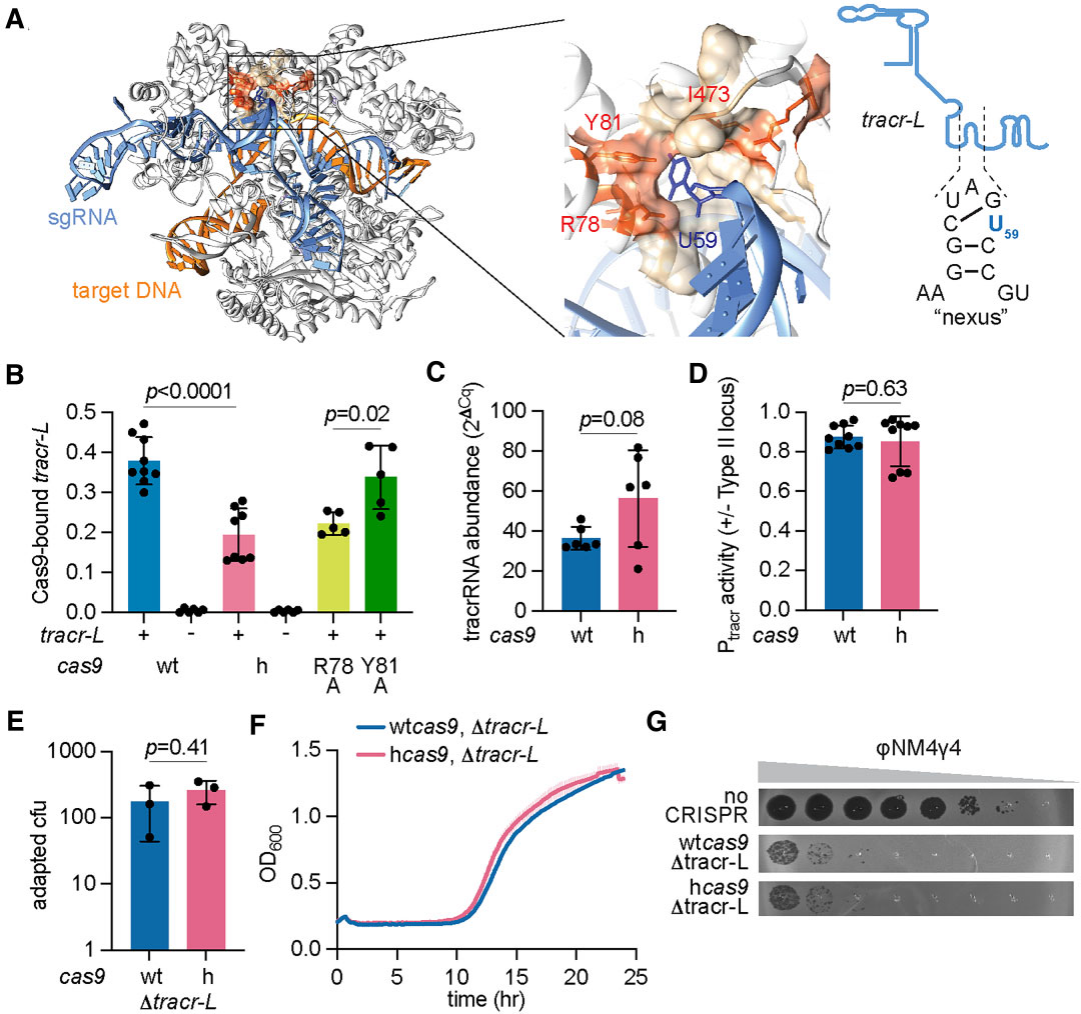
The I473F mutation decreases binding of Cas9 to *tracr-L* to relieve repression of *cas* expression and enhance the type II-A response. (**A**) Structure of Cas9 (PDB: 4UN3) highlighting the hydrophobic pocket lined by I473, R78 and Y81 that makes contacts with an uracil base (U59) present in the nexus hairpin of the tracrRNA. A schematic and sequence of the *tracr-L* nexus is shown on the right. (**B**) RNA quantification of *tracr-L* bound to wtCas9 or hCas9 via qPCR normalized to bound crRNA. Cas9-bound RNA was isolated from FLAG pulldowns of FLAG-tagged versions of wtCas9 or hCas9. Means of three biological replicates (three technical replicates each) ± SD are reported. For Δlong, R78A and Y81A samples, means of a minimum of five technical replicates ± SD are reported. (**C**) Quantification of tracrRNA via qRT-PCR normalized to housekeeping gene *rho*. Total RNA was isolated from overnight *S. aureus* cultures harboring pCRISPR containing wtCas9 or hCas9 (I473F). Means of six technical replicates ± SD are reported. (**D**) Promoter activity was measured as the fluorescence/OD_600_ ratio in cells harboring a plasmid expressing GFP from P*tracr-L* (promoter for *tracr-L*) and a second plasmid encoding pCRISPR with wtCas9 or hCas9. Promoter activity was normalized to an empty vector control. Means of three biological replicates (three technical replicates each) ± SD are reported. (**E**) Plasmids harboring spacer-less CRISPR-Cas arrays containing a 52-bp deletion spanning P*tracr-L* and 19 nucleotides at the 5′ end of *tracr-L* (Δlong) were infected with ϕNM4γ4 (MOI∼2) on top agar. Surviving colonies with expanded CRISPR arrays were quantified by PCR. Mean of three biological replicates ± SD are reported. (**F**) Growth of *S. aureus* expressing pCRISPR Δlong (strains from (**C**)) upon infection with ϕNM4γ4 (MOI ∼10), measured as the OD_600_ of the cultures over time. Mean of three biological replicates ± SEM are reported. (**G**) Detection of plaque formation after spotting 10-fold serial dilutions of ϕNM4γ4 on top agar seeded with *S. aureus* expressing pCRISPR Δlong or carrying an empty vector control. Plasmids were programmed with a spacer in the CRISPR array to target a region of the viral genome followed by a NAG PAM. Representatives of three biological replicates shown.

As mentioned above, the tracrRNA locus of the *S. pyogenes* type II-A CRISPR-*cas* system can be transcribed from two different promoters, leading to the production of two versions of the Cas9 cofactor of different lengths, *tracr-L* and *tracr-S* (Figure 1A). *Tracr-L* functions as a natural single guide RNA that represses *cas* gene expression by directing Cas9 to bind P*cas* (19) (Figure 1A). Given that residue I473 is located in a pocket that accommodates a hairpin within the tracrRNA known as the nexus (21) (Figure 3A), we hypothesized that the mutation of this residue to phenylalanine could affect Cas9 interaction with its cofactor in a manner that reduces the repression of the P*cas* promoter. To test this, we compared the ability of wtCas9 and hCas9 to bind *tracr-L* by tagging the nucleases with a 3xFLAG-tag, pulling down the proteins, extracting the bound RNA from an equivalent amount of protein and measuring *tracr-L* abundance via RT-qPCR. The CRISPR array of these tagged strains contained the first natural spacer present in *S. pyogenes* (*spc1*, Figure 1A) to ensure the generation of *spc1* crRNA, which Ct values were used to normalize the abundance of bound *tracr-L*. As a control, we deleted the *tracr-L* promoter in the plasmids harboring the tagged version of wt*cas9* and h*cas9*. No RT-qPCR signal for *tracr-L* was detected after pull-downs from these strains; however, we found that wtCas9 was associated with approximately two-times more *tracr-L* than hCas9 (Figure 3B). One possible explanation for this result is that the I473F mutation somehow reduces the levels of tracrRNA available for hCas9 binding. To test this, we used RT-qPCR to measure the abundance of the Cas9 cofactor present in total RNA extracts of staphylococci carrying wt*cas9* or h*cas9*. We detected a small but not statistically significant increase in the tracrRNA levels in cells expressing hCas9 (Figure 3C). In addition, we introduced a plasmid carrying *gfp* fused to the *tracr-L* promoter into strains harboring wt*cas9* or h*cas9*, and measured green fluorescence. We found similar levels of GFP signal (Figure 3D), a result that, together with the data presented in Figure 3C, indicates that the abundance of *tracr-L* does not decrease in staphylococci expressing hCas9.

We reasoned that if the increase in hCas9 expression is due to a deficiency in binding *tracr-L* that prevents repression of the P*cas* promoter, mutations that abrogate recognition of P*cas* by this RNA should mimic the phenotypes associated with the I473F substitution in cells expressing wtCas9. To test this hypothesis, we first tested a deletion of the *tracr-L* promoter (which results in the absence of *tracr-L* associated with wtCas9 and hCas9, Figure 3B) and measured spacer acquisition and NAG-targeting. We found that, after infection with ϕNM4γ4, both the number of CRISPR-resistant colonies (Figure 3E) as well as the ability of cultures to recover from infection (Figure 3F), were improved in the strain expressing wt*cas9*, showing similar results to those obtained in the h*cas9* backgrounds, both with and without the deletion of the *tracr-L* promoter (see results in Figure 2A, B). Similarly, the targeting of an NAG-flanked sequence in ϕNM4γ4 also improved in the strain expressing wtCas9 but not *tracr-L*, resulting in a similarly low phage propagation as cells harboring hcas9 (Figure 3G). Finally, we eliminated the repression of *tracr-L* by introducing a single mutation in the P*cas* promoter that eliminates the PAM (GGG to GGC, Supplementary Figure S2A) required for its interaction with Cas9 (19). The mutation was introduced in plasmids carrying both wt*cas9* and h*cas9*, and the respective strains were infected with ϕNM4γ4 to determine the ability of the cultures to acquire new spacers against this phage and regrow. Similarly to the results obtained for the cultures in which *tracr-L* was not produced, staphylococci carrying the P*cas* mutation and expressing wtCas9 displayed a strong survival curve, equivalent to that of cells expressing hCas9 (Supplementary Figure S2B).

The original characterization of the I473F mutation showed that hCas9 and wtCas9 have similar cleavage properties of targets containing NGG or NAG PAMs *in vitro* (20). In these experiments, Cas9 was loaded with *in vitro*-transcribed *tracr-S* and crRNA molecules prior to incubation with a target DNA. These results suggest that the I473F mutation does not interfere with the binding and function of *tracr-S*. If this is the case, hCas9 should be able to repress P*cas* expression using *tracr-S* and a crRNA that directs the enzyme to the promoter. To test this, we designed a spacer that produces a crRNA guide with 11 nucleotides that are complementary to the P*cas* region recognized by *tracr-L* (the other 9 nucleotides that compose the 20-nucleotide guiding sequence do not have a match; Supplementary Figure S2C). The spacer was then cloned into pCRISPR plasmids expressing wtCas9 or hCas9 that contain a deletion of the *tracr-L* promoter, to ensure expression of *tracr-S* only. The plasmids were introduced into a strain harboring the P*cas*-*gfp* reporter and green fluorescence was measured to determine promoter activity (Supplementary Figure S2D). We found equally low levels of green fluorescence in cultures expressing wtCas9 and hCas9, similar to wtCas9 in the presence of *tracr-L* (Figure 1C) and we therefore conclude that the I473F mutation does not impact the function of *tracr-S*. Altogether, these data demonstrate that the phenotypes associated with the I473F mutation in Cas9 are a consequence of a defective binding of *tracr-L* that prevents proper repression of the P*cas* promoter that is mediated by the longer version of the tracrRNA, and thus results in an enhanced expression of the type II-A *cas* genes.

### A Cas9 pocket that interacts with the tracrRNA nexus can modulate *cas* gene expression

**Figure 4.**
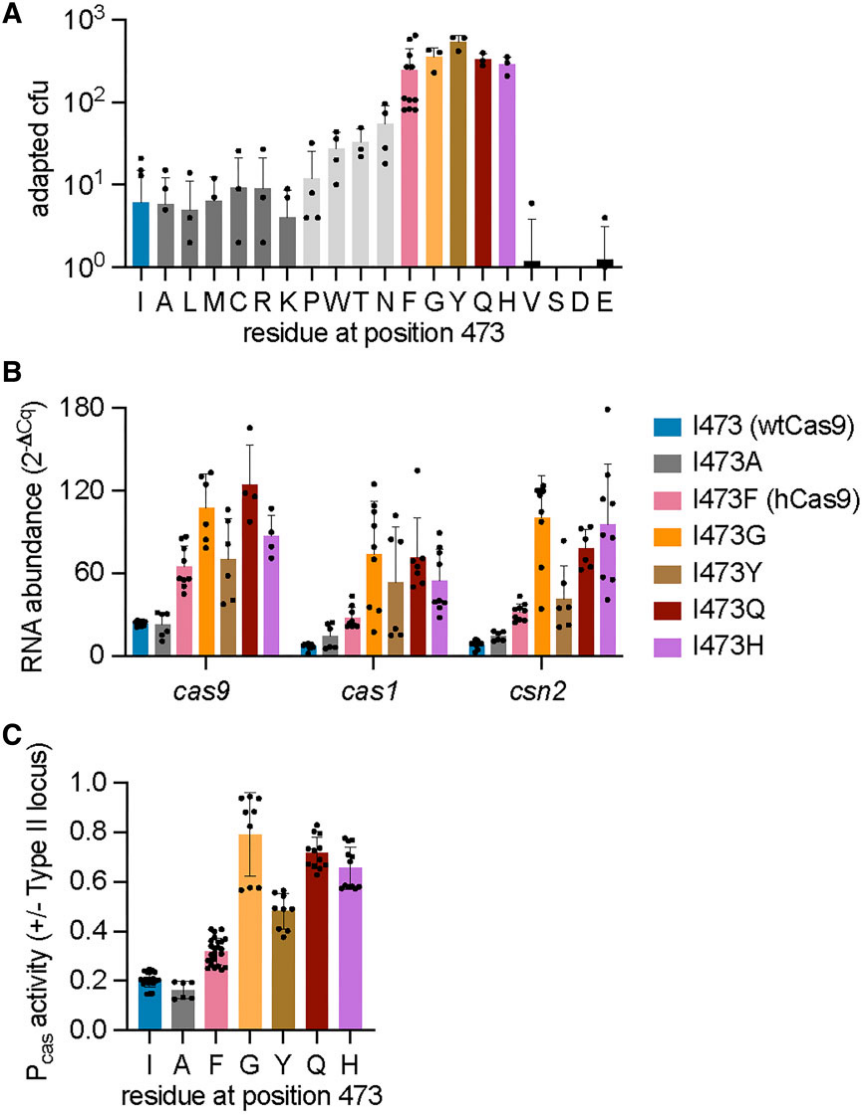
Cas9 amino acids at position 473 can modulate *cas* gene expression. (**A**) Staphylococci carrying plasmids with spacer-less CRISPR-Cas arrays and expressing different Cas9 mutants harboring amino acids substitutions at position 473 of the Cas9 protein were infected with ϕNM4γ4 (MOI∼2) on top agar. Surviving colonies with expanded CRISPR arrays were quantified by PCR. Adaption for I473S and I473D was below the limit of detection. Means of a minimum of three biological replicates ± SD are reported. (**B**) RNA quantification of *cas* gene transcripts via qRT-PCR normalized to housekeeping gene *rho*. Total RNA was isolated from overnight *S. aureus* cultures carrying plasmids that express different Cas9 I473 mutants. Means of three biological replicates ± SD are reported. (**C**) Promoter activity was measured as the fluorescence/OD_600_ ratio in cells harboring a reporter plasmid expressing GFP from P*cas* and a second plasmid encoding pCRISPR with select Cas9 I473 mutations. Promoter activity was normalized to an empty vector control. Means of a minimum of three technical replicates ± SD are reported.

We wondered whether other amino acid chains beyond phenylalanine at position 473 could also affect the P*cas* repression mediated by *tracr-L*. To investigate this, we mutated I473 to all other amino acids and measured the efficiency of spacer acquisition through the enumeration of colonies that form after infection with ϕNM4γ4. We found that substitutions to glycine, tyrosine, glutamine, and histidine phenocopied the I473F mutation (Figure 4A and Supplementary Figure S3A). In contrast, mutations to alanine, leucine, methionine, cysteine, arginine and lysine maintained a wild-type phenotype (Figure 4A and Supplementary Figure S3B). Substitutions to proline, tryptophan, tyrosine and asparagine displayed an intermediate level of spacer acquisition (Figure 4A). Finally, mutations to valine, serine, aspartate and glutamate severely disrupted type II-A immunity, reducing the number of CRISPR-resistant colonies to almost undetectable levels (Figure 4A). To test whether the gain of function observed for the glycine, tyrosine, glutamine, and histidine substitutions is a result of enhanced transcription, we measured the abundance of the *cas9*, *cas1* and *cas2* transcripts using RT-qPCR and compared the values to those obtained for the alanine substitution. We found that all of these mutations, but not I473A, increased transcription of the *cas* genes, in some cases to higher levels than those observed for cells expressing hCas9 (Figure 4B). These results were corroborated by transforming the *gfp* reporter plasmid (Figures 1C and 2D) into staphylococci carrying these substitutions and quantifying green fluorescence as an alternative measure of P*cas* activity (Figure 4C). Although these results did not reveal a particular type of amino acid side chain that is required to relieve the repression of the *tracr-L* on P*cas*, our data supports a model in which the residue at position 473 is key for the interaction between Cas9 and tracrRNA in the context of the autoregulation of type II-A *cas* gene expression.

**Figure 5.**
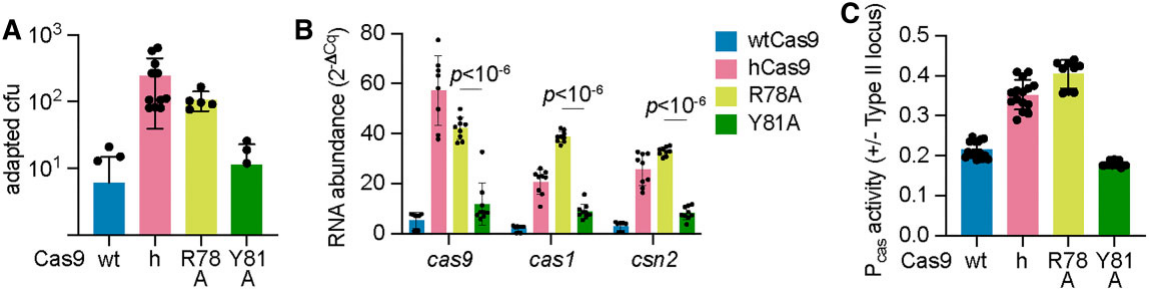
A Cas9 pocket that interacts with the tracrRNA nexus can modulate *cas* gene expression. (**A**) Plasmids harboring spacer-less CRISPR-Cas arrays and expressing wtCas9, hCas9, Cas9^R78A^ or Cas9^Y81A^ were infected with ϕNM4γ4 (MOI∼2) on top agar. Surviving colonies with expanded CRISPR arrays were quantified by PCR. Means of a minimum of five biological replicates ± SD are reported. (**B**) RNA quantification of *cas* gene transcripts via qRT-PCR normalized to housekeeping gene *rho*. Total RNA was isolated from overnight *S. aureus* cultures harboring pCRISPR plasmids with the indicated Cas9 mutation. Means of three biological replicates (three technical replicates each) ± SD are reported. (**C**) Promoter activity was measured as the fluorescence/OD_600_ ratio in cells harboring a reporter plasmid expressing GFP from P*cas* and a second pCRISPR plasmid encoding select Cas9 mutants. Promoter activity was normalized to an empty vector control. Means of three biological replicates (three technical replicates each) ± SD are reported.

We also investigated the impact of other residues in the vicinity of I473. This isoleucine is located within a hydrophobic pocket of the Cas9 recognition lobe that makes base-specific contacts with a uracil base (U59) present in the nexus hairpin of the tracrRNA (36) (Figure 3A and Supplementary Figure S4A). In addition to I473, this pocket contains two other side chains, R78 and Y81, that contact U59, and therefore we mutated each of these to alanine and evaluated the resulting Cas9 mutants. First, we looked at spacer acquisition and found that the R78A mutation caused an increase in colonies that survive ϕNM4γ4 infection via spacer acquisition similar to that of hCas9 (Figure 5A and Supplementary Figure S4B). In contrast, the Y81A substitution retained wild-type levels of colony formation and spacer acquisition (Figure 5A and Supplementary Figure S4B). The enhancement in spacer acquisition of the R78A Cas9 mutant was correlated with an increase in *cas* gene expression, measured both by RT-qPCR (Fig. 5B) and green fluorescence using the P*cas*-*gfp* reporter plasmid (Figure 5C). Interestingly, the ability of both mutant Cas9 nucleases to recognize a NAG-containing target and prevent the propagation of ϕNM4γ4 was similar to that of wtCas9 (Supplementary Figure S4C). This result suggests that the R78A mutation can affect not only Cas9 function as a repressor of P*cas* but also Cas9 dsDNA cleavage activity. Given this unexpected result, we decided to measure the binding of *tracr-L* to the mutant Cas9 enzymes. We added a 3xFLAG-tag to the R78A and Y81A variants, pulled down the proteins, extracted the bound RNA from an equivalent protein amount and measured *tracr-L* abundance via RT-qPCR. Consistent with the results obtained for spacer acquisition and *cas* gene expression, we found lower levels of *tracr-L* associated with the R78A mutant, similar to those measured for hCas9 (Fig. 3B). In contrast, the amount of *tracr-L* bound to the Y81A mutant was higher, similar to that of wtCas9. These data support our model in which the Cas9 pocket that interacts with the nexus hairpin of the tracrRNA is important for the proper function of this nuclease in the repression of transcription from the P*cas* promoter.

### Cas9^I473F^ enhances adaptation in other type II-A systems that produce a *tracr-L* isoform

**Figure 6.**
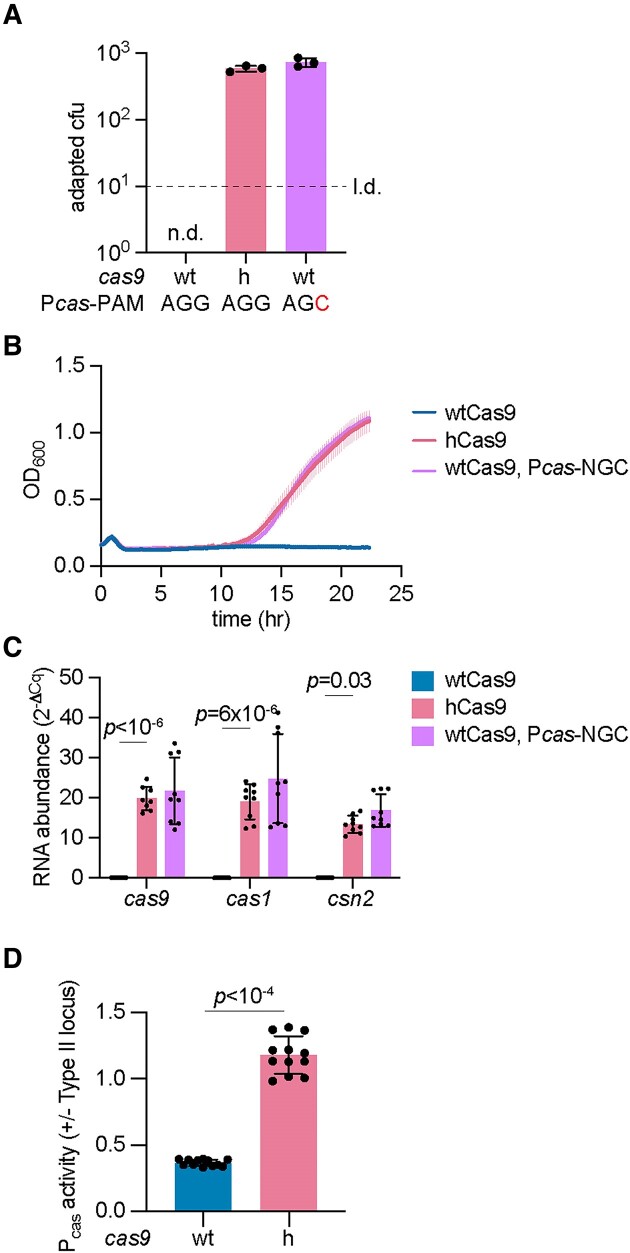
The I473F mutation in Cas9 enhances spacer acquisition in the *S. mutans* type II-A CRISPR-Cas system. (**A**) Plasmids harboring the *S. mutans* type II-A CRISPR-Cas system with wtCas9, hCas9 or wtCas9 with a P*cas* PAM mutation (AGG to AGC) were infected with ϕNM4γ4 (MOI∼2) on top agar. Surviving colonies with expanded CRISPR arrays were quantified by PCR. Adaptation for wtCas9 was not detected (n.d.) within the limit of detection (l.d.). Means of three biological replicates ± SD are reported. (**B**) Growth of *S. aureus* expressing *S. mutans* pCRISPR upon infection with ϕNM4γ4 (MOI ∼10), measured as the OD_600_ of the cultures over time. Mean of three biological replicates ± SEM are reported. (**C**) RNA quantification of *cas* gene transcripts via qRT-PCR normalized to housekeeping gene *rho*. Total RNA was isolated from overnight *S. aureus* cultures harboring the pCRISPR plasmids shown in (A). Means of three biological replicates ± SD are reported. (**D**) Promoter activity was measured as the fluorescence/OD_600_ ratio in cells harboring a reporter plasmid expressing GFP from *S. mutans* type II-A P*cas* promoter and a second pCRISPR plasmid expressing wtCas9 or hCas9. Promoter activity was normalized to an empty vector control. Means of three biological replicates (three technical replicates each) ± SD are reported.

Finally, we investigated if our findings can be expanded to other type II-A CRISPR-Cas systems. We chose to study the *Streptococcus mutans* NN2025 Cas9 enzyme due to (i) its conservation of I473 and the adjacent amino acids (Supplementary Figure S5A), and (ii) that sequence analysis suggested the presence of an operator sequence in the P*cas* promoter of the *S. mutans* type II-A CRISPR-Cas system that could be repressed by *tracr-L* (Supplementary Figure S5B). The P*cas* region of *S. pyogenes* and *S. mutans* shared 66% identity, however in the latter the *tracr-L* target seems to be located downstream of the -10 promoter element (Supplementary Figure S5C). Based on our previous results, these predictions indicate that the I473F substitution in *S. mutans* Cas9 should increase the frequency of spacer acquisition. To test this hypothesis, we cloned this CRISPR-Cas system into the shuttle vector pLZ12 (37) and measured spacer acquisition by enumerating colonies that form after ϕNM4γ4 infection. The number of CRISPR-resistant colonies derived from cells harboring h*Cas9* was improved by nearly 3 logs relative to cells harboring wt*Cas9* (Figure 6A and Supplementary Figure S5D). To compare this phenotype with that of a strain that lacks repression, we mutated the putative PAM of the *tracr-L* target in the P*cas* promoter of the *S. mutans* type II-A CRISPR locus (AGG to AGC, Supplementary Figure S5B). Staphylococci carrying this mutation caused an increase in colonies that survive ϕNM4γ4 infection via spacer acquisition similar to that of hCas9 (Figure 6A and Supplementary Figure S5D). We also followed the re-growth of cultures expressing both wtCas9 and hCas9 and found that the I473F mutation led to a faster recovery of the infected cells (Figure 6B). After infection of staphylococci carrying the P*cas* PAM mutation with ϕNM4γ4, we observed a rapid regrowth, almost identical to that of cultures expressing hCas9 and a wild-type P*cas* promoter (Figure 6B). To corroborate that, as it is the case for the *S. pyogenes* type II CRISPR-Cas system, the I473F mutation leads to higher transcription of the *cas* genes, we performed RT-qPCR. Indeed, our measurements indicated higher levels of *cas9*, *cas1* and *csn2* in cells expressing hCas9, similar to those observed in cells carrying the P*cas*-AGC mutation (Figure 6C). These results were corroborated by transforming a reporter plasmid harboring the *gfp* gene under the control of the *S. mutans* P*cas* promoter into staphylococci carrying the I473F substitution; cultures expressing hCas9 displayed higher levels of green fluorescence (Figure 6D). Altogether, these results indicate that the I473F mutation displays similar effects in related Cas9 variants.

## Discussion

Here, we investigated the mechanism by which the I473F mutation in Cas9 enhances spacer acquisition and immunity against phage target sequences harboring a suboptimal NAG PAM. We found that the mutation affects the binding of *tracr-L* to Cas9, leading to a decrease in the repression of the *cas* genes. Overexpression of the *cas* operon has been previously shown to increase spacer acquisition (19,23,24) and we believe that it is conceivable that higher concentrations of Cas9 in the cell would improve the recognition and cleavage of targets containing NAG PAMs. This is because the affinity of Cas9 for NAG PAMs has been determined to be ∼20 times less than for the NGG sequence (38) and therefore an increase of the Cas9 levels would raise the availability of the nuclease for the targets with suboptimal PAMs.

Residue I473 is located within a hydrophobic pocket of the Cas9 recognition lobe that interacts with the nexus loop of the tracrRNA (Figure 3A) (36). While this RNA hairpin is present in both *tracr-L* and *tracr-S* (Supplementary Figure S4A), our data suggests that its interaction with the I473 pocket is more critical for the binding and/or function of the *tracr-L* form. In addition to poor binding, the I473F substitutions could also impair the proper positioning of the upstream region of *tracr-L* that is necessary for the recognition of the P*cas* target sequence. This hypothesis is supported by previous findings that showed that hCas9 and wtCas9 have similar cleavage properties of targets containing NGG or NAG PAMs *in vitro* (20) when the nucleases are associated with *tracr-S* and crRNA. In addition, our results demonstrated that hCas9 is able to repress *cas* gene expression when it is loaded with *tracr-S* and a crRNA guide that specifies the *tracr-L* target sequence within the P*cas* promoter (Supplementary Figure S2D). Both of these results suggest that the I473F mutation does not interfere with the binding and function of *tracr-S*. Perhaps in the case of *tracr-L*, since the tracrRNA and the guide RNA are connected in the same molecule, changes in the binding of the tracrRNA nexus could be transmitted to the guide RNA region and affect its ability to recognize its target, P*cas*. On the other hand, in the case of *tracr-S*, the cofactor and guide RNAs are independent molecules and therefore changes in the binding of *tracr-S* cannot be transmitted to the crRNA and thus do not affect recognition of the target. The perturbations caused by the changes in position 78 within this pocket have a less predictable effect, since the R78A substitution displayed higher levels of immunity against phage targets with NAG PAMs than wtCas9 (but not as much as hCas9; compare Figure 2D and Supplementary Figure S4C) in spite of the presence of a higher level of the *cas9* transcript (Figure 5B), and presumably of the Cas9 nuclease, in the infected cells. Therefore, it is possible that this mutation affects both the repressor abilities of *tracr-L* and the function of Cas9^R78A^ to cleave its targets using *tracr-S* and a crRNA guide.

We did not find a clear pattern for the I473 substitutions that phenocopy the mutation to phenylalanine (Figure 4). While substitution of I473 for tyrosine displayed similar properties to the I473F change, I473W only increased spacer acquisition to intermediate levels, suggesting other factors beyond steric hindrance are involved. Similar results were obtained with substitutions to glutamine (which phenocopied I473F) and to asparagine (intermediate levels). Surprisingly, the change to glycine also resulted in hCas9 levels of spacer acquisition. This is most likely due to the absence of specific contacts or hydrogen bonds between I473 and the uracil nucleobase (U59) positioned within the Cas9 pocket (Figure 3A) (36). To put these results in an evolutionary context, we aligned 1000 Cas9 sequences and found a high conservation of isoleucine in position 473 (79.8% of the sequences, Supplementary Figure S6A). From the residues that we found to maintain wild-type levels of spacer acquisition (Figure 4A), alanine and leucine are often present in Cas9 homologs (each present in 2% of the homologs). In contrast, valine was found to be highly conserved in position 473 (13% of the homologs) but it is very detrimental for CRISPR adaptation (Figure 4A). On the other hand, residues at position 473 that increase spacer acquisition and *cas* gene expression (phenylalanine, tyrosine, glycine and histidine; Figure 4A–C) were not detected in the alignment (Supplementary Figure S6A), suggesting an evolutionary disadvantage for the overexpression of the type II-A *cas* operon (20). Alignment results showed an even higher conservation at position 78 (Supplementary Figure S6B), with 97.9% of Cas9 homologs carrying arginine and most of the others harboring also positively charged residues such as lysine (1.9%) and histidine (0.1%). Alanine, the residue that we found can increase *cas* gene expression when located in position 78, was not found in Cas9 homologs, an observation that also suggests an evolutionary disadvantage for elevated frequencies of spacer acquisition. In contrast, position 81 displayed no strong amino acid conservation (Supplementary Figure S6C), a result that is in line with our observations that this position is less critical for modulation of the P*cas* promoter via *tracr-L* (Figure 5A–C).

We made similar observations for the I473F mutation in a related *S. mutans* type II-A CRISPR-Cas system, which harbors a similar Cas9 nuclease but a divergent *tracr-L* and P*cas* target (Supplementary Figure S5). Therefore, our results suggest that the interaction between the I473 pocket and the nexus hairpin is critical for the appropriate regulation of type II-A *cas* genes in strains that produce the *tracr-L* form (19). Given that expression of hCas9 has a fitness cost for the host cell (presumably due to the higher levels of self-spacer acquisition) (20), it is conceivable that other regions of Cas9 have evolved to tightly bind *tracr-L* to ensure its repressor function and prevent the negative side effects of increased spacer acquisition frequency and minimize collateral damage to the host population. Further characterization of Cas9 residues that interact with different *tracr-L* sequences will yield insights into the evolution of the transcriptional regulation of type II CRISPR-Cas systems.

## Supplementary Material

gkae597_Supplemental_File

## Data Availability

The data underlying this article are available in the article and in its online supplementary material.
