# Supplementary material for: Cas9 interaction with the tracrRNA nexus modulates the repression of type II-A CRISPR-cas genes
Source: Nucleic Acids Res. 2024 Jul 12;52(17):10595–606. doi: 10.1093/nar/gkae597 (PMC11417352; doi:10.1093/nar/gkae597)
Supplement: gkae597_Supplemental_File [file gkae597_supplemental_file.pdf]

Cas9 interaction with the tracrRNA nexus modulates the repression of type II-A CRISPR-*cas* genes

Hyejin Kim[1,3] and Luciano A. Marraffini[1,2,*]

[1]Laboratory of Bacteriology, The Rockefeller University, 1230 York Ave, New York, NY 10065, USA.

[2]Howard Hughes Medical Institute, The Rockefeller University, 1230 York Ave, New York, NY 10065, USA.

[3]Weill Cornell/Rockefeller/Sloan Kettering Tri-Institutional MD-PhD Program, New York, NY, USA.

*Corresponding author: marraffini@rockefeller.edu

SUPPLEMENTARY FIGURES

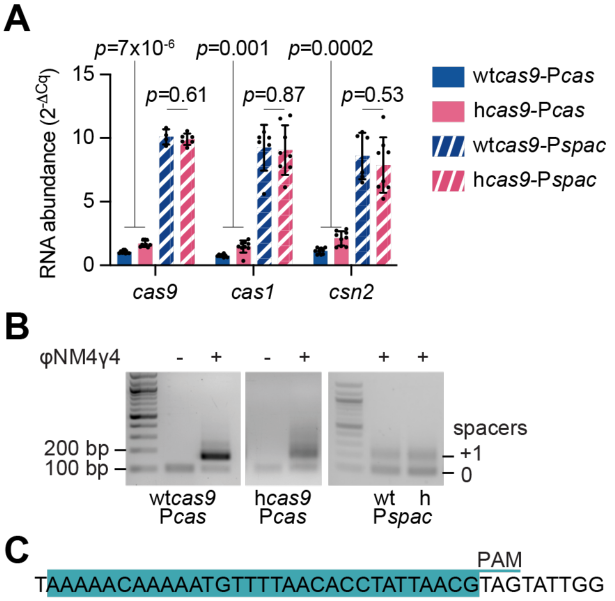

Supplementary Figure 1. *cas* gene expression under the control of P*spac* is similar for wild type Cas9 and hCas9. (A) RNA quantification of *cas* gene transcripts via qRT-PCR normalized to housekeeping gene *rho*. Total RNA was isolated from early log phase *S. aureus* cultures harboring pCRISPR expressing wild-type Cas9 or hCas9 with *cas* gene expression under the control of native P*cas* or constitutive P*spac* promoters. Means of three biological replicates + SD are reported. **(B)** Agarose gel electrophoresis of PCR products obtained after amplification of the CRISPR array using DNA obtained from infections with ϕNM4γ4 (MOI10) in Figure 1D. **(C)** Sequence of the ϕNM4γ4 target used in Figure 1G, containing a NAG protospacer adjacent motif (PAM).

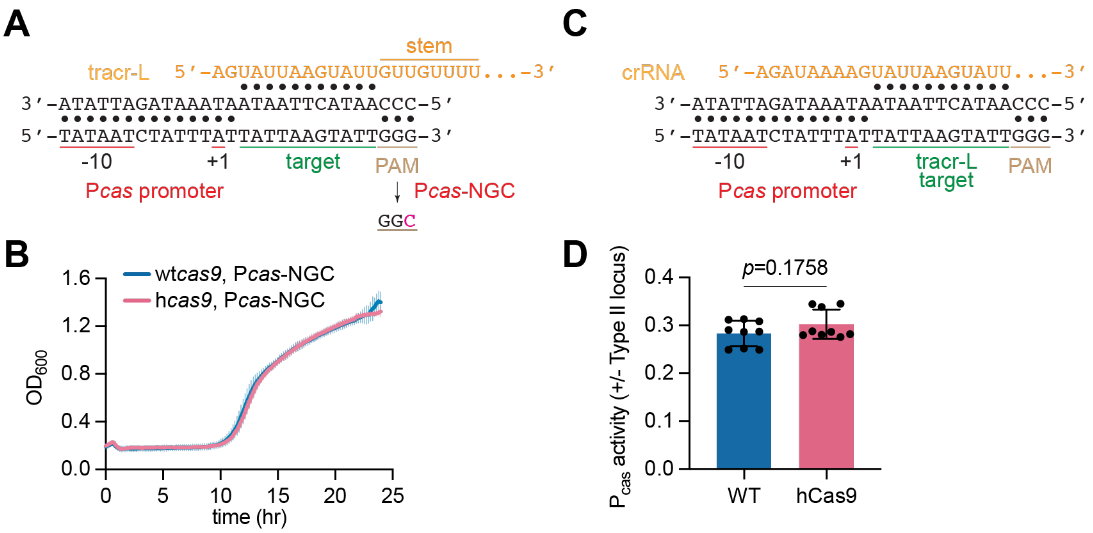

Supplementary Figure 2. Mutation of the **P*cas* target PAM relieves *tracr-L*-mediated *cas* repression to enhance the type II-A response. (A)** Schematic of *tracr-L*-mediated repression of the P*cas* promoter in *S. pyogenes*. Highlighted in green is the 11-nucleotide match between the 5' end of *tracr-L* and the P*cas* promoter. The putative GGG PAM of the P*cas* target was mutated to GGC. **(B)** Growth of *S. aureus* harboring a pCRISPR with a P*cas* PAM mutation, GGC, upon infection with ϕNM4γ4 (MOI ~10), measured as the OD_{600} of the cultures over time. Mean of three biological replicates $\pm$ SEM are reported. **(C)** Schematic of *tracr-S*/crRNA-mediated repression of the P*cas* promoter in *S. pyogenes*. Highlighted in green is the 11-nucleotide match between the 5' end of crRNA and the P_{cas} promoter. **(D)** P*cas* promoter activity measured as the green fluorescence/OD_{600} ratio in cells harboring a plasmid harboring a P*cas*-*gfp* reporter and a second plasmid encoding pCRISPR with wild-type Cas9 or hCas9 in the presence of a deletion of the tracr-L promoter (only *tracr-S* is present in the cells). Promoter activity was normalized to an empty vector control. Means of five biological replicates (3 technical replicates each) $\pm$ SD are reported.

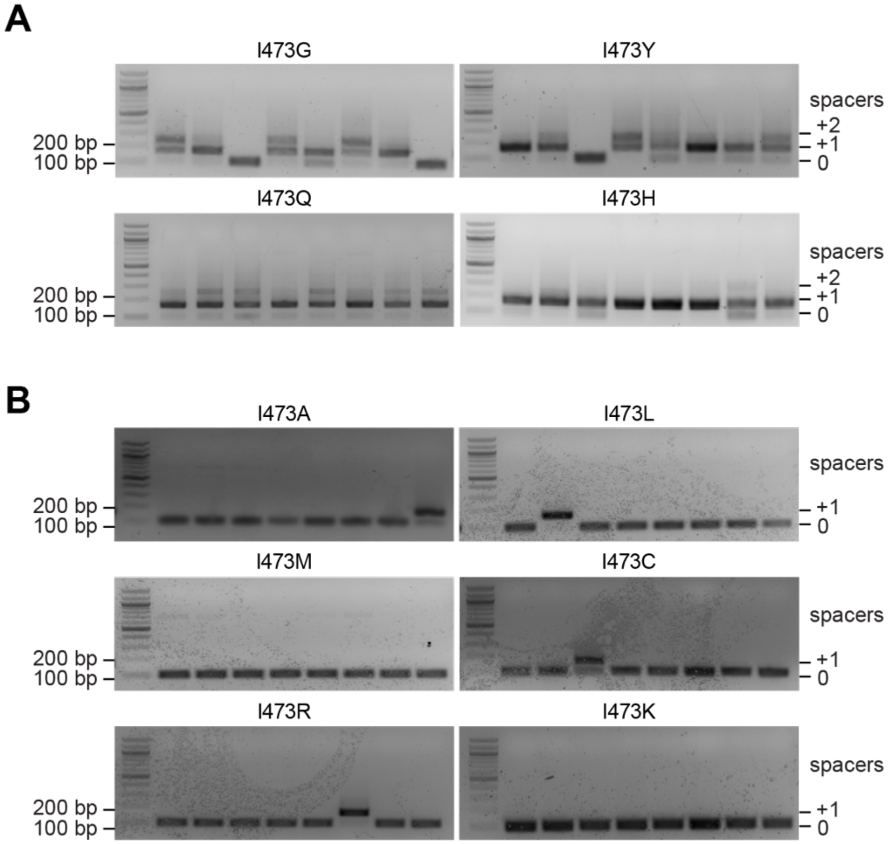

Supplementary Figure 3. Mutations in Cas9 residue 473 can modulate P*cas* repression. (A) Agarose gel electrophoresis of PCR products obtained after amplification of the CRISPR array using DNA obtained from colonies formed on top agar, after infection with ϕNM4γ4 (MOI~2), from the experiment shown in Figure 4A with I473 substitutions that enhance spacer acquisition. **(B)** Same as **(A)** but testing I473 substitutions that do not alter spacer acquisition frequency.

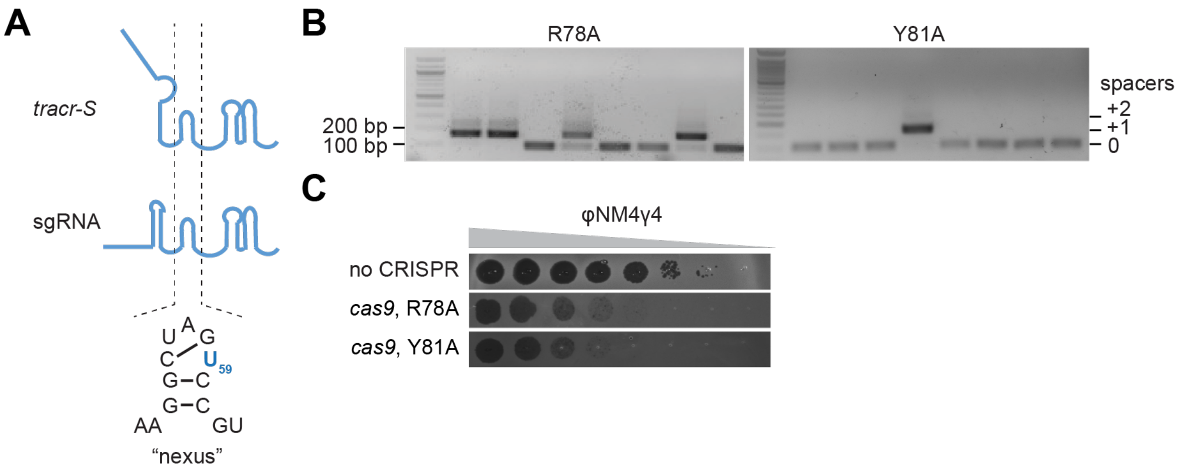

Supplementary Figure 4. The Cas9 pocket interacting with tracrRNA nexus is important for P*cas* repression. (A) Schematic of the tracrRNA nexus shared by *tracr-S* and sgRNA. The highlighted U59 base of the tracrRNA nexus is predicted to interact with the Cas9 pocket displayed in Figure 3A. **(B)** Agarose gel electrophoresis of PCR products obtained after amplification of the CRISPR array using DNA obtained from colonies formed on top agar that express $Cas9^{R78A}$ or $Cas9^{Y81A}$, after infection with ϕNM4γ4 (MOI~2), from the experiment shown in Figure 5A. **(C)** Detection of plaque formation after spotting 10-fold serial dilutions of ϕNM4γ4 phage on top agar seeded with *S. aureus* expressing $Cas9^{R78A}$ or $Cas9^{Y81A}$ or an empty vector control. Plasmids were programmed with a spacer in the CRISPR array to target a region of the viral genome followed by a NAG PAM. Representatives of three biological replicates shown.

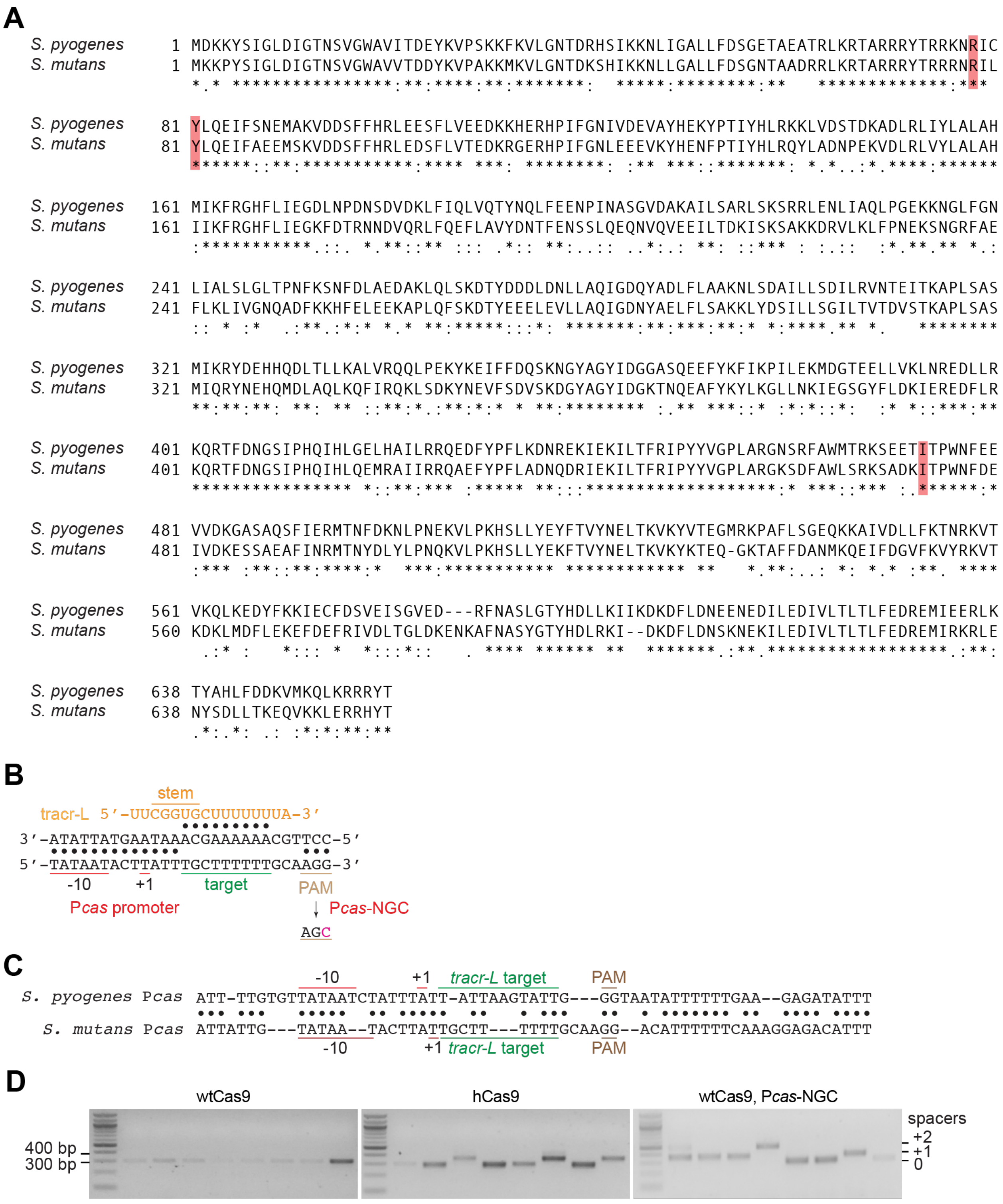

Supplementary Figure 5. $Cas9^{I473F}$ enhances spacer acquisition in other type II-A systems that produce a *tracr-L* isoform. (A) TCOFFEE alignment of the Cas9 proteins encoded by *S. pyogenes* SF370 and *S. mutans* NN2025. Highlighted in red are the residues of interest that have been mutated in this study. (*) indicates identical residues. (:) indicates strongly similar residues. (.) indicates weakly similar residues. **(B)**

Schematic of *tracr-L*-mediated repression of the P*cas* promoter in *S. mutans*. Highlighted in green is the 9-nucleotide match between the 3' end of *tracr-L* and the P*cas* promoter. The putative AGG PAM of the P*cas* target was mutated to AGC in Figure 6. **(C)** Alignment of the P*cas* region of *S. pyogenes* and *S. mutans*. **(D)** Agarose gel electrophoresis of PCR products obtained after amplification of the CRISPR array using DNA obtained from colonies formed on top agar after infection with ϕNM4γ4 (MOI~2), in the experiment described in Figure 6A.

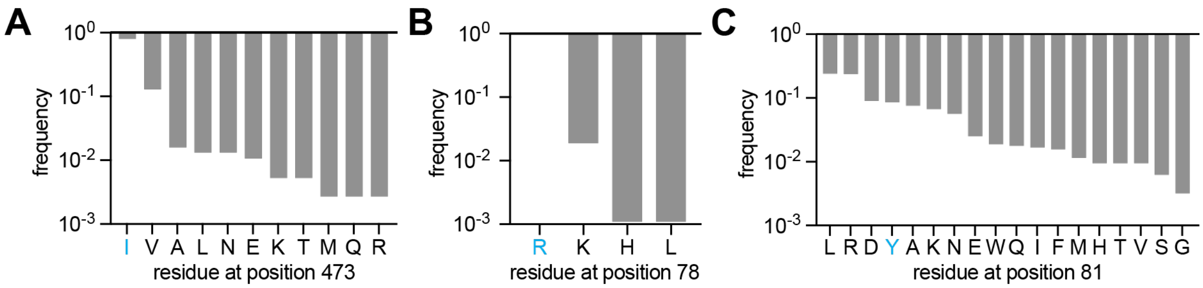

Supplementary Figure 6. Aligment of 1,000 Cas9 protein sequences. (A) Frequency of conservation of amino acid residues at position 473 of *S. pyogenes* SF370 Cas9 (the residue present in this protein is noted in blue). **(B)** Same as **(A)** but for position 78. **(C)** Same as **(A)** but for position 81.

SUPPLEMENTARY TABLES

Supplementary Table 1. Plasmids used in this study.

Plasmid	Description	Reference
pC194	Cloning vector	(1)
pE194	Cloning vector	(2)
pLZ12	Cloning vector	(3)
pDB114	Cloning vector	(4)
pLG29	*S. pyogenes* P_{cas}-GFP reporter	(5)
pLG3	*S. pyogenes* $P_{tracr\text{-}L}$-GFP reporter	(5)
pGG32	*S. pyogenes* pCRISPR with native array	(6)
pRH065	*S. pyogenes* pCRISPR targeting with spc65	(7)
pRH087	*S. pyogenes* pCRISPR with 1st native spacer	(7)
pRH163	hCas9 in GG32 backbone	(7)
pHK30	pGG32 Δtracr-L	This study
pHK31	pRH163 Δtracr-L	This study
pHK34	hCas9 in RH087 backbone	This study
pHK41	3xFLAG WT Cas9 in RH087 backbone	This study
pHK42	3xFLAG hCas9 in HK34 backbone	This study
pHK58	Mutated P_{cas} PAM to NGC in pGG32	This study
pHK59	Mutated P_{cas} PAM to NGC in pRH163	This study
pHK78	hCas9 in RH065 backbone	This study
pHK132	3xFLAG WT Cas9 Δtracr-L	This study
pHK133	3xFLAG hCas9 Δtracr-L	This study
pHK171	GG32 with cas genes under control of P_{spac}	This study
pHK175	RH163 with cas genes under control of P_{spac}	This study
pHK180	RH065 Δtracr-L	This study
pHK181	HK78 Δtracr-L	This study
pHK184	*S. pyogenes* pCRISPR targeting with spc64	This study
pHK185	hCas9 in HK184 backbone	This study
pHK191	RH065 with Cas9 under control of P_{spac}	This study
pHK192	HK78 with hCas9 under control of P_{spac}	This study
pHK211	3xFLAG Cas9^{R78A} in RH087 backbone	This study
pHK212	3xFLAG Cas9^{Y81A} in RH087 backbone	This study

pHK218	Cas9^{R78A} in RH065 backbone	This study
pHK219	Cas9^{Y81A} in RH065 backbone	This study
pJM75	*S. mutans* pCRISPR in pC194	This study
pHK230	*S. mutans* pCRISPR in LZ12	This study
pHK232	*S. mutans* pCRISPR I473F in LZ12	This study
pHK233	*S. mutans* pCRISPR Pcas-ngc in LZ12	This study
pHK323	*S. mutans* P_{cas}-GFP reporter in LZ12 with SpecR replaced by CmR	This study

1. Horinouchi, S. and Weisblum, B. (1982) Nucleotide sequence and functional map of pC194, a plasmid that specifies inducible chloramphenicol resistance. *J. Bacteriol.*, **150**, 815-825.

2. Horinouchi, S. and Weisblum, B. (1982) Nucleotide sequence and functional map of pE194, a plasmid that specifies inducible resistance to macrolide, lincosamide, and streptogramin type B antibodies. *J. Bacteriol.*, **150**, 804-814.

3. Perez-Casal, J., Caparon, M.G. and Scott, J.R. (1991) Mry, a trans-acting positive regulator of the M protein gene of *Streptococcus pyogenes* with similarity to the receptor proteins of two-component regulatory systems. *J. Bacteriol.*, **173**, 2617-2624.

4. Bikard, D., Euler, C.W., Jiang, W., Nussenzweig, P.M., Goldberg, G.W., Duportet, X., Fischetti, V.A. and Marraffini, L.A. (2014) Exploiting CRISPR-Cas nucleases to produce sequence-specific antimicrobials. *Nat. Biotechnol.*, **32**, 1146-1150.

5. Workman, R.E., Pammi, T., Nguyen, B.T.K., Graeff, L.W., Smith, E., Sebald, S.M., Stoltzfus, M.J., Euler, C.W. and Modell, J.W. (2021) A natural single-guide RNA repurposes Cas9 to autoregulate CRISPR-Cas expression. *Cell*, **184**, 675-688 e619.

6. Heler, R., Samai, P., Modell, J.W., Weiner, C., Goldberg, G.W., Bikard, D. and Marraffini, L.A. (2015) Cas9 specifies functional viral targets during CRISPR-Cas adaptation. *Nature*, **519**, 199-202.

7. Heler, R., Wright, A.V., Vucelja, M., Bikard, D., Doudna, J.A. and Marraffini, L.A. (2017) Mutations in Cas9 Enhance the Rate of Acquisition of Viral Spacer Sequences during the CRISPR-Cas Immune Response. *Mol. Cell*, **65**, 168-175.

Supplementary Table 2. Oligonucleotides used in this study.

Name	Sequence	Purpose
HK1	GGAAGTCTGAAGAAACACTTACCCCATGGAATTTTG	Cas9 I473L
HK2	CAAAATTCCATGGGGTAAGTGTTTCTTCAGACTTCC	Cas9 I473L
HK3	GGAAGTCTGAAGAAACAGTTACCCCATGGAATTTTG	Cas9 I473V
HK4	CAAAATTCCATGGGGTAACTGTTTCTTCAGACTTCC	Cas9 I473V
HK5	GGAAGTCTGAAGAAACAACTACCCCATGGAATTTTG	Cas9 I473T
HK6	CAAAATTCCATGGGGTAGTTGTTTCTTCAGACTTCC	Cas9 I473T
HK7	GGAAGTCTGAAGAAACAAATACCCCATGGAATTTTG	Cas9 I473N
HK8	CAAAATTCCATGGGGTATTTGTTTCTTCAGACTTCC	Cas9 I473N
HK9	GGAAGTCTGAAGAAACAAGTACCCCATGGAATTTTG	Cas9 I473S
HK10	CAAAATTCCATGGGGTACTTGTTTCTTCAGACTTCC	Cas9 I473S
HK11	GGAAGTCTGAAGAAACAAAGACCCCATGGAATTTTG	Cas9 I473K
HK12	CAAAATTCCATGGGGTCTTTGTTTCTTCAGACTTCC	Cas9 I473K
HK13	GGAAGTCTGAAGAAACAAGAACCCCATGGAATTTTG	Cas9 I473R
HK14	CAAAATTCCATGGGGTTCTTGTTTCTTCAGACTTCC	Cas9 I473R
HK15	GGAAGTCTGAAGAAACATATACCCCATGGAATTTTG	Cas9 I473Y
HK16	CAAAATTCCATGGGGTATATGTTTCTTCAGACTTCC	Cas9 I473Y
HK17	GGAAGTCTGAAGAAACATGTACCCCATGGAATTTTG	Cas9 I473C
HK18	CAAAATTCCATGGGGTACATGTTTCTTCAGACTTCC	Cas9 I473C
HK19	GGAAGTCTGAAGAAACATGGACCCCATGGAATTTTG	Cas9 I473W
HK20	CAAAATTCCATGGGGTCCATGTTTCTTCAGACTTCC	Cas9 I473W
HK21	GGAAGTCTGAAGAAACACCTACCCCATGGAATTTTG	Cas9 I473P

HK22	CAAAATTCCATGGGGTAGGTGTTTCTTCAGACTTCC	Cas9 I473P
HK23	GGAAGTCTGAAGAAACACATACCCCATGGAATTTTG	Cas9 I473H
HK24	CAAAATTCCATGGGGTATGTGTTTCTTCAGACTTCC	Cas9 I473H
HK25	GGAAGTCTGAAGAAACACAAACCCCATGGAATTTTG	Cas9 I473Q
HK26	CAAAATTCCATGGGGTTTGTGTTTCTTCAGACTTCC	Cas9 I473Q
HK27	GGAAGTCTGAAGAAACAGATACCCCATGGAATTTTG	Cas9 I473D
HK28	CAAAATTCCATGGGGTATCTGTTTCTTCAGACTTCC	Cas9 I473D
HK29	GGAAGTCTGAAGAAACAGAAACCCCATGGAATTTTG	Cas9 I473E
HK30	CAAAATTCCATGGGGTTTCTGTTTCTTCAGACTTCC	Cas9 I473E
HK31	GGAAGTCTGAAGAAACAGGTACCCCATGGAATTTTG	Cas9 I473G
HK32	CAAAATTCCATGGGGTACCTGTTTCTTCAGACTTCC	Cas9 I473G
HK33	GGAAGTCTGAAGAAACAATGACCCCATGGAATTTTG	Cas9 I473M
HK34	CAAAATTCCATGGGGTCATTGTTTCTTCAGACTTCC	Cas9 I473M
HK44	GGAAGTCTGAAGAAACAGCTACCCCATGGAATTTTG	Cas9 I473A
HK45	CAAAATTCCATGGGGTAGCTGTTTCTTCAGACTTCC	Cas9 I473A
HK38	AGGATCATGATGGTGATTATAAAGATCACGACATCGATTACAAAGATGATGACGATAAAGATAAGAAATACTCAATAGGCTTAGATATCG	3xFLAG Cas9
HK39	CGTGATCTTTATAATCACCATCATGATCCTTGTAGTCCATTTTTGCCTCCTAAAAT	3xFLAG Cas9
HK42	CTTTCTCAAGTTATCATCGGCAATG	*S. pyogenes* array
HK100	AGTGCGATTACAAAATTTTTTAGAC	*S. pyogenes* array
HK95	TGCTGTTACTTTAAGACTTACAACAGAAG	pE194 cloning
HK96	CTTCTGTTGTAAGTCTTAAAGTAACAGCA	pE194 cloning
HK105	TATTAAGTATTGGCTAATATTTTTTGAAGAGATATTTTG	$P_{cas\text{-}NGC}$
HK106	CAAAATATCTCTTCAAAAAATATTAGCCAATACTTAATA	$P_{cas\text{-}NGC}$
HK146	TCTGACTTCCGAAAAGATTTCC	qPCR (Cas9)
HK147	GCAGTTCCAACGACGGCA	qPCR (Cas9)

HK148	GAACCATTTAGGCCTTTAGTGG	qPCR (Cas1)
HK149	CATATCCTAAATTCAGGAACTCCTTTC	qPCR (Cas1)
HK150	CCATTAAGAGGCGGTACAATTC	qPCR (Csn2)
HK151	CCTCATATTGGTAACAATATTGCAC	qPCR (Csn2)
HK159	GCATCTGGTTTGTTCGTACTGATTG	qPCR (rho)
HK160	CCAGATGAGCGTATTAAATTAGAGACAG	qPCR (rho)
HK161	CTTTTATAACAAATAATCAAGGAGAAATTC	qPCR (tracr-L)
HK162	GTATTAAGTATTGTTTTATGGCTGATAAAT	qPCR (tracr-L)
HK155	GGTGCCACTTTTTCAAGTTGATAAC	qPCR (tracr-S)
HK200	TGGAACCATTCAAAACAGCATAGCA	qPCR (tracr-S)
HK165	GTTTTAGAGCTATGCTGTTTTGAATG	qPCR (crRNA)
HK166	AAAAGCGCAAGAAGAAATCAAC	qPCR (crRNA)
HK196	TATTTGAACCAACAAACGACTTTTAGTATAACC	pC194 cloning
HK197	GGTTATACTAAAAGTCGTTTGTTGGTTCAAATA	pC194 cloning
HK233	TTATTGCTTTTTTGCAAGCACATTTTTTCAAAGGA	*Smut* $P_{cas\text{-}NGC}$
HK234	TCCTTTGAAAAAATGTGCTTGCAAAAAAGCAATA A	*Smut* $P_{cas\text{-}NGC}$
HK249	ATACACGTCGGAAGAATGCTATTTGTTATCTACA GGAG	SpyCas9 R78A
HK250	CTCCTGTAGATAACAAATAGCATTCTTCCGACGT GTAT	SpyCas9 R78A
HK251	ATACACGTCGGAAGAATCGTATTTGTGCTCTACA GGAG	SpyCas9 Y81A
HK252	CTCCTGTAGAGCACAAATACGATTCTTCCGACGT GTAT	SpyCas9 Y81A
HK265	TTTATCTACAAGGTGTGGCATAATGTGTGGAATT GTGAGCGGATAACAATTGTAATATTTTTGAAGA GATATTTTGAAAAAGAAA	P_{spac}-cas
HK266	TGTTATCCGCTCACAATTCCACACATTATGCCAC ACCTTGTAGATAAAGTCAAACACAAAATTCTTTTA AAAAGTAGTTTATTTTG	P_{spac}-cas
HK275	TTTTTAAAAGAATTTTGTGTTATAATCTATTTATTA TTAAGTATTGGGT	Δtracr-L
HK276	TTATAACACAAAATTCTTTTAAAAATGGCTGATAA ATTTCTTTGAATTTCTCC	Δtracr-L
HK286	ATCGGCTGATAAATTTACACCATGGAAT	SmCas9 I473F
HK287	ATTCCATGGTGTAAATTTATCAGCCGAT	SmCas9 I473F
HK215	GCTTTGGCACATATAATTAAGTTTAGAGG	*Smut* qPCR (Cas9)
HK220	ATTCTTGAAACAGTCTTTGTACATCATTATTGC	*Smut* qPCR (Cas9)
HK221	GCTAAACAATCAGATTTCTTGGGCAG	*Smut* qPCR (Cas1)
HK222	GAACATTCTCCCAAATAACATGACTGAT	*Smut* qPCR (Cas1)
HK225	TGGTTCACGCTGATTTAGAAAATCAGT	*Smut* qPCR (Csn2)

HK226	GCAATTAATTCCGTAATGGTATTTGCC	*Smut* qPCR (Csn2)
HK219	AACAAGAAAAGCGCTAGAAAGATTGATTTCTA	*Smut* array
HK347	TGAGCGCAACGCAATTAA	*Smut* array
JM386	AAAACCTACAGAAAACACTAAATTAATAAGAAAGAGCCAAACCTCGAAAG	*Smut* pCRISPR cloning
JM389	GGCTCTTTCTTATTAATTTAGTGTTTTCTGTAGGTTTTTAGGCATAAAACTATATG	*Smut* pCRISPR cloning
JM511	GCTAGTAAACCGCCTCGCGCAGCTTTTAAAAAGCAAATATGAGCC	*Smut* pCRISPR cloning
JM512	GGCTCATATTTGCTTTTTAAAAGCTGCGCGAGGCGGTTTACTAGC	*Smut* pCRISPR cloning
HK391	AAACACTAAATTAATAAGAAAGAGCCAAACC	*Smut* pCRISPR cloning
HK392	GCGCGAGGCGGTTTACTA	*Smut* pCRISPR cloning
HK393	TGCCCGCTAGTAAACCGCCTCGCGCGGTCATAACCTGAAGGAAGATCTGG	*Smut* pCRISPR cloning
HK394	GCTCTTTCTTATTAATTTAGTGTTTATCTGTGCCAGTTCGTAATGTCTG	*Smut* pCRISPR cloning
HK493	TGTTTCCACCATTTTTTCAATTTTTCACTTTAGATAAAAATTTAGGAGGCATATCAA	LZ12 SpecR→CmR
HK494	AAAAAATAACCTTATTGGTACTTACTTATAAAAGCCAGTCATTAGGCCTATCT	LZ12 SpecR→CmR
HK495	GTAAGTACCAATAAGGTTATTTTTTAAATGTTTCC	LZ12 SpecR→CmR
HK496	AAAAATTGAAAAAATGGTGGAAACACTTTT	LZ12 SpecR→CmR
